# Occurrence of Priming in the Degradation of Lignocellulose in Marine Sediments

**DOI:** 10.1371/journal.pone.0143917

**Published:** 2015-12-03

**Authors:** Evangelia Gontikaki, Barry Thornton, Thomas Cornulier, Ursula Witte

**Affiliations:** 1 Institute of Biological and Environmental Science, Oceanlab, University of Aberdeen, Newburgh, United Kingdom; 2 The James Hutton Institute, Aberdeen, United Kingdom; 3 Institute of Biological and Environmental Science, University of Aberdeen, St. Machar Drive, Aberdeen, United Kingdom; Auckland University of Technology, NEW ZEALAND

## Abstract

More than 50% of terrestrially-derived organic carbon (terrOC) flux from the continents to the ocean is remineralised in the coastal zone despite its perceived high refractivity. The efficient degradation of terrOC in the marine environment could be fuelled by labile marine-derived material, a phenomenon known as “priming effect”, but experimental data to confirm this mechanism are lacking. We tested this hypothesis by treating coastal sediments with ^13^C-lignocellulose, as a proxy for terrOC, with and without addition of unlabelled diatom detritus that served as the priming inducer. The occurrence of priming was assessed by the difference in lignocellulose mineralisation between diatom-amended treatments and controls in aerobic sediment slurries. Priming of lignocellulose degradation was observed only at the initial stages of the experiment (day 7) and coincided with overall high microbial activity as exemplified by total CO_2_ production. Lignocellulose mineralisation did not differ consistently between diatom treatments and control for the remaining experimental time (days 14–28). Based on this pattern, we hypothesize that the faster initiation of lignocellulose mineralisation in diatom-amended treatments is attributed to the decomposition of accessible polysaccharide components within the lignocellulose complex by activated diatom degraders. The fact that diatom-degraders contributed to lignocellulose degradation was also supported by the different patterns in ^13^C-enrichment of phospholipid fatty acids between treatments. Although we did not observe differences between treatments in the total quantity of respired lignocellulose at the end of the experiment, differences in timing could be important in natural ecosystems where the amount of time that a certain compound is subject to aerobic degradation before burial to deeper anoxic sediments may be limited.

## Introduction

Understanding the fate of carbon delivered from the continents to the ocean is critical for constraining Earth system models and predicting human-induced effects on global biogeochemical cycles. Ecosystems along the land-ocean continuum play a pivotal role in terrestrially-derived organic carbon (terrOC) transformation, sequestration and export to the open ocean [1,2]; ~55–80% of dissolved and particulate OC flux from the continents is remineralised in the coastal zone, the remainder being preserved, buried and reincorporated into the longer-term components of the sedimentary cycle [3,4]. The apparent high remineralisation efficiency of terrOC in the coastal ocean comes into contrast with the presumed refractory nature of land-derived material. This disparity has been described as one of the major “conundrums” in oceanography [5,6]_,_ however the mechanisms behind terrOC processing at the land-ocean interface remain major unknowns in coastal carbon research [7]. Recently, Guenet et al.[8] highlighted the priming effect as a possible major process enabling the efficient removal of terrOC from aquatic and marine ecosystems. Priming effects refer to short-term changes, usually an increase, in the microbial degradation of refractory organic matter upon addition of labile material and have been described in soils. Recent studies have confirmed that bulk organic matter in marine sediments is also subject to priming [9,10]. Regarding terrOC in particular, priming “hotspots” could arise in zones where terrOC and labile autochthonous marine organic matter (marOC) co-occur, such as river plumes and estuarine/coastal sediments [6].

TerrOC entering marine ecosystems derives largely from vascular plant cell wall polymers, collectively referred to as lignocellulose [6]. Lignocellulose is a highly versatile composite of three complex biopolymers; microfibrils of cellulose are linked by less-ordered polysaccharides (hemicellulose) and embedded in lignin, a complex and heterogeneous phenolic macromolecule. As one of the most abundant reserves of fixed carbon on the planet, the degradation of lignocellulose underpins the wider function of ecosystems and carbon cycling at a global scale. However, the tertiary architecture of lignocellulose and the highly resistant coating of lignin around the polysaccharide network constitute a physical and chemical barrier to degradation. Nevertheless a number of microorganisms have developed the capability to utilise this nutrient source using a wide range of hydrolytic and oxidative enzymes [11,12] and the rate and extent of lignocellulose degradation in a particular environment depends on the presence and activity of these specialist microorganisms [13,14]. Numerous studies have demonstrated the immediate response of benthic microbial communities to the deposition of algal-derived material, characterised by high rates of oxidation of highly labile carbon immediately after amendment followed by the slower utilisation of less labile material [15–18]. Much less is known however about the life strategies and response times of lignocellulose degraders in marine ecosystems.

Lignocellulose degradation on land is thought to be driven by fungi [19] but it is unclear if these microorganisms perform equivalent roles in the marine environment. Marine fungi appear to be rare and less diverse than their terrestrial counterparts [20]. Several species of marine fungi however possess the ability to produce lignocellulose-degrading enzymes and are thought to play a significant role in the degradation of large lignocellulosic substrates, such as decaying salt marsh grass and mangrove wood [19,21]. On the other hand, the broader physiological versatility of bacteria allow them to occupy ecological niches at environmental conditions considered unfavourable to fungi and due to their unicellular form, they are better adapted for growth on small particulate material even at low nutrient concentrations [22]. It has thus been suggested that the relative importance of bacteria as plant-derived organic matter degraders might be greater in aquatic systems where most terrOC is in particulate or dissolved form and is degraded in the water column and sediments under waterlogged conditions [23,24]. This idea is supported by the widespread presence and high abundance of marine bacterial genera capable of recycling plant cell wall polysaccharides (*Microbulbifer*-*Teredinibacter-Saccharophagus*) [25,26] and of indigenous Actinomycetes in marine sediments that, similarly to terrestrial strains, may play important roles in the degradation of vascular plant-derived biopolymers, including lignin [27–29]. Despite recent advances, we have probably discovered only a tiny fraction of the diversity of marine lignocellulose degraders which hampers a full understanding of the fate of terrOC in the marine environment.

The importance of interactions between labile and less biodegradable pools of OC and their effect on the balance between organic matter degradation and preservation in sediments have long been recognised [30]. Nevertheless, only a handful of studies have attempted to assess the occurrence and magnitude of priming in the degradation of bulk sediment organic carbon [9,10,18] and the hypothesis that this phenomenon may explain the fast degradation of terrOC in the marine environment [6,8] has not yet been tested experimentally. Here, we provide the first experimental test of this hypothesis using a novel dual-substrate approach that allows direct estimation of the effects of priming thus decreasing statistical errors. Specifically, we treated coastal sediments with ^13^C-labelled lignocellulose, as a proxy for particulate terrOC, and followed its degradation with and without addition of unlabelled marine diatom detritus that served as the priming inducer. We hypothesised that lignocellulose degradation, expressed as ^13^CO_2_ production would be greater in diatom-amended treatments compared to controls due to priming and that the magnitude of the effect of priming would be positively correlated to diatom quantity. In addition, we traced the fate of lignocellulosic carbon into microbial biomass by analysis of ^13^C uptake into phospholipid fatty acid (PLFA) biomarkers, which allowed following the dynamics of different groups of microorganisms within the lignocellulose-responsive microbial population. To our knowledge, this is the first study to provide experimental data on the effect of priming on terrestrially-derived organic matter degradation in marine sediments and capture changes in the structure of benthic lignocellulose-responsive communities depending on the level of marOC input.

## Materials and Methods

### Sediment collection and experimental setup

Sediment was collected from mudflats in the lower reach of the Ythan estuary, Aberdeenshire, Scotland, UK (57° 20.085’N, 02° 0.206’W) in October 2011. All necessary permissions for work on the Ythan and Forvie National Nature Reserve were obtained from Scottish Natural Heritage. The sediment at the sampling location was muddy sand (mean particle size = 50.0 *μ*m, silt content 60.0%) and contained 1.5% organic carbon by dry weight in the upper 1 cm. The carbon to nitrogen ratio was 7.3. Prior to incubation, the sediment was sieved over a 500 *μ*m mesh to remove macrofauna and was allowed to settle for 24 h to retain the fine fraction. The effect of sieving on the biomass, community structure and metabolic status of sediment microbial communities has been shown to be short-term and non-detectable 8 to 24 h after the disturbance [31]. Sediment slurries were prepared in 125 mL amber glass vials, each vial containing 20 mL of sediment, 20 mL of 10 *μ*m-filtered, UV-sterilized seawater collected from the estuary at high tide and a substrate or combination of substrates depending on the treatment: ^13^C-lignocellulose only (^13^C-CTRL), ^13^C-lignocellulose + x amount of unlabelled diatoms (1D), ^13^C-lignocellulose + 2x amount of unlabelled diatoms (2D). Incubations without any substrate addition were used to determine background mineralisation rates (CTRL treatment). The highly labelled lignocellulose used in the experiments (97 atom% ^13^C; IsoLife, The Netherlands) was obtained from wheat by sulphur-free soda pulping followed by further purification through selective extraction steps. Subsequent purity analysis after hydrolysis was performed by High-Performance Anion-Exchange Chromatography (Thermo Scientific™ Dionex™). The ^13^C-labelled lignocellulose had a C/N ratio of 107 and a polysaccharide content of 76.5%.

The quantity of ^13^C-lignocellulose was the same in all treatments and equivalent to 0.15 mg C mL^-1^ wet sediment. This quantity was calculated based on lignin concentrations in Loch Etive and Loch Creran (0.65 mg lignin products/100 mg OC [32]), the OC content at the sampling station and an average lignin percentage in lignocellulose from wheat of 18% [11,33]. Freeze-dried biomass of the diatom *Thalassiosira pseudonana* Hasle & Heimdal was used as the priming inducer at quantities equivalent to 0.23 and 0.47 mg C mL^-1^ wet sediment for 1D and 2D treatments respectively (3 and 6% of the total OC content in sediments). These values fall below and above the total amount of phytoplankton that would be deposited on Ythan sediments throughout the duration of the incubation period (mean daily deposition calculated at 137 mg C m^-2^ d^-1^; deposition of phytoplankton detritus in sediments is assumed to amount to 50% of the mean annual phytoplankton production in the Ythan estuary [34]). Details of algal culture and harvest techniques are presented elsewhere [16].

Sediment slurries were incubated for 28 days with sampling intervals at 7, 14 and 21 days. Due to destructive sampling, a different set of incubation vials was prepared for each sampling time (3 replicates per treatment and sampling time, *n* = 48). Initial conditions were determined from a separate set of vials (*n* = 3), which were sampled immediately after preparation. The incubation vials were kept in a temperature-controlled room at the average temperature for the Ythan estuary in October (10°C) and were shaken manually daily. Oxygen was monitored using a needle-type oxygen probe (Firesting, Pyroscience) and air in the headspace of the incubation vials was exchanged weekly through the PTFE/silicone septum-fitted screw caps with a mixture of 80% N_2_: 20% O_2_ (BOC) to maintain aerobic conditions. The vials were purged with the gas mixture for 10 min and the total CO_2_ produced during each sampling interval was captured in two successive NaOH traps (2 M). At each sampling interval, a subset of vials was opened and a water sample was drawn and sterile-filtered (0.2 *μ*m) into Exetainers (Labco, U.K.) for analysis of dissolved inorganic carbon concentration and isotopic enrichment (DIC and DI^13^C). The remaining sediment was stored at -20°C for subsequent quantification of lignocellulosic carbon assimilation into microbial biomass (based on PLFA biomarkers).

### Sample analysis

The concentration and isotopic signature of CO_2_ in both gas and liquid phases were measured on a Gas-bench II connected to a Delta^Plus^ Advantage isotope ratio mass spectrometer (IRMS; both Thermo Finnigan, Germany). DIC samples were quantitatively converted to CO_2_ by acidification before analysis. CO_2_ concentrations were calculated from the combined area counts of masses 44, 45 and 46 given in the standard output of the IRMS and a calibration curve derived from known DIC concentration standards [35]. PLFAs were extracted from ~ 3 g of freeze-dried sediment using a single phase extractant consisting of chloroform, methanol and citrate buffer (1:2:0.8 v/v/v) for 2 h following a modified Bligh and Dyer protocol [36]. The total lipid extract was fractionated into polarity classes on silicic acid columns (6 mL ISOLUTE SIS PE columns, International Sorbent Technology Ltd, UK) by sequential elution with chloroform (neutral lipids), acetone (glycolipids) and methanol (phospholipids). PLFAs were transmethylated under alkaline methanolysis to yield fatty acid methyl esters (FAME). The concentration and carbon isotope ratios of individual FAMEs were measured on a GC Trace Ultra with combustion column attached via a GC Combustion III to a Delta V Advantage IRMS (all Thermo Finnigan, Germany) [37].

### Calculations

Carbon isotopes were calculated using ISODAT NT software version 2.0 (ThermoElectron) and are expressed in the delta notation (δ ‰) relative to a reference according to:

δC13(‰)=[(Rsample/RVPDB)–1]×1000 (1)

where *R*_*sample*_ and *R*_*VPDB*_ is the ^13^C:^12^C ratios of the sample and the international reference material for carbon (Vienna Pee Dee Belemnite; VPDB) respectively (*R*_*VPDB*_ = 0.0112372). The δ^13^C of total CO_2_ (δ^13^C_total_) in the vials was calculated as the concentration-weighed average of that measured in the headspace and water after 1‰ correction for fractionation between CO_2_ gas in the aqueous and gaseous phase [38] as:

δCtotal13=(δ13Cgas×[CO2]gas)+(δ13Cwater×[CO2]water)[CO2]gas+[CO2]water (2)

Lignocellulose respiration was calculated as the product of excess ^13^C (*E*) and the total CO_2_ concentration in the vial (sum of CO_2_ in gas and water phase), divided by the fractional abundance of ^13^C in lignocellulose (97 atom% ^13^C) [39]. Excess ^13^C (*E*) is given by the difference in the fraction ^13^C in ^13^C-CTRL, 1D and 2D treatments (*F*_*sample*_) and background controls at each time point (*F*_*background*_):

$$E=F_{sample}–F_{background}$$ (3)

where F=C13C+C1213=RR+1 and R=(δC131000+1)×RVPDB

Excess ^13^C within each biomarker PLFA was calculated by Eq 3. Incorporation of lignocellulosic carbon into individual PLFAs (I_PLFA_) was calculated as the product of *E* and the concentration of each PLFA divided by the fractional abundance of ^13^C in lignocellulose [40]. The PLFAs i15:0, a15:0, i16:0 and 18:1ω7 were used as bacterial biomarkers and C18 PUFA and 18:1ω9 as biomarkers for microeukaryotes [41]. The concentration and ^13^C enrichment of the biomarker PLFAs provided a relative estimate of biomass and lignocellulosic carbon uptake by the respective microbial groups.

### Statistical analysis

The effect of time and treatment (both categorical variables) on total and lignocellulose mineralisation, and biomass of bacteria and microeukaryotes (total and enriched biomass expressed as the sum of concentrations and enrichment of biomarker PLFAs respectively) was tested using linear regression. Due to the time effect being defined as a categorical variable, this regression model is equivalent to type 1 ANOVA. A model validation was applied to check that the underlying statistical assumptions were not violated: homogeneity of variance was evaluated by plotting the residuals vs. the fitted values, normality was assessed by plotting the theoretical quantiles vs. the standardised residuals (qq plots) and independence was evaluated by plotting the residuals vs. each explanatory variable. No data transformation was necessary. Post-hoc multiple comparisons were carried out using Tukey’s honest significant difference test.

The structure of the lignocellulose-degrading community as a function of time and treatment was examined using partial least squares regression (PLS; package “plsdepot”) [42] on the proportional ^13^C-enrichment of individual PLFAs (I_PLFA_/I_total_). PLS is particularly suitable when a regression is needed to explain the structure of a multivariate signal, such as multiple PLFA enrichments, with a (possibly large) set of covariates. To normalize the response data we applied an empirical logit transform with a tolerance of 0.1 to the PLFA relative enrichments. PLFAs with a mean relative enrichment <0.001 were not included in the analysis. PLS was also used to compare the enriched PLFA profiles from this study (response variables) with published PLFA profiles of known or suspected lignocellulose-degrading bacteria or cultured representatives of bacterial genera that include strains encoding plant cell wall-degrading enzymes in their genome (explanatory variables). The PLFA database included profiles of 82 bacterial isolates belonging to 24 genera and 4 phyla. The PLFA profiles of bacterial isolates within a genus were averaged so that the dataset used in PLS consisted of 24 explanatory variables (predictor matrix: 14 rows of individual PLFAs ☓ 24 columns of bacterial genera). Only common PLFAs between the bacterial isolates dataset and enriched profiles from this study were included in the analysis. The analysis was initially performed using all combinations of treatment ☓ time as response variables (response matrix: 14 rows of individual PLFAs ☓ 12 columns of treatment and time combinations). The results did not suggest a temporal pattern with treatment and the analysis was repeated with enriched PLFA profiles averaged over time for clarity of presentation (response matrix with three columns corresponding to each treatment). All statistical analyses were conducted in the “R” programming environment [43].

## Results

### Effect of diatom detritus addition on mineralisation

**Fig 1:**
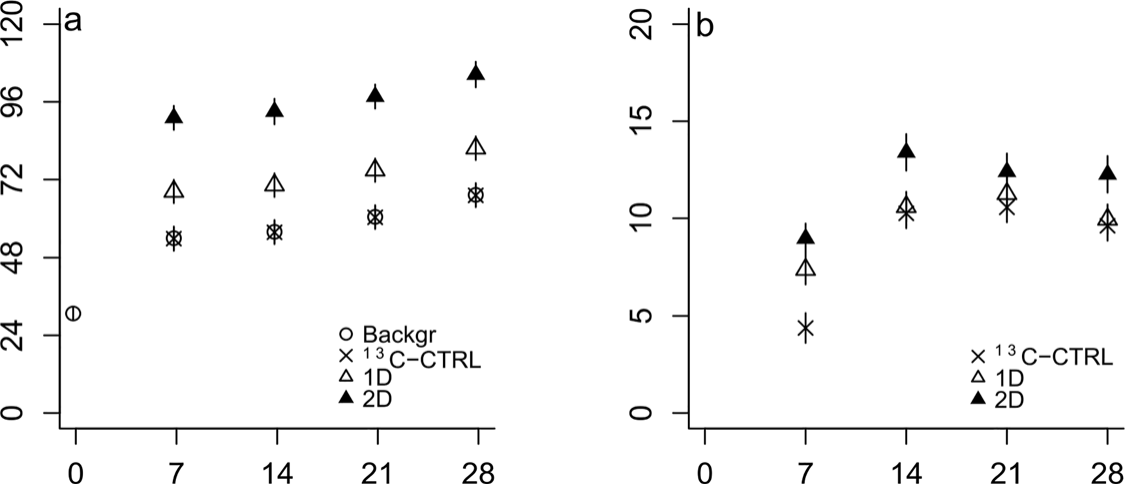
Total respiration (a) and respiration of lignocellulosic carbon (b) with time (μg C mL^-1^wet sediment). Data from CTRL (background mineralisation), ^13^C-CTRL (^13^C-lignocellulose) and dual substrate treatments 1D and 2D (^13^C-lignocellulose + unlabelled diatoms) are represented by diagonal crosses, circles, and open/filled triangles respectively. Values are mean ± SE (*n* = 3).

Diatom quantity caused a significant, step-wise increase of total CO_2_ production (*F* = 141.66, d.f._3, 39_, *p*<0.001) during the first week of the experiment (Fig 1A). Respiration rates dropped to similar levels in all treatments between days 7 and 28 (as shown by the identical slopes of CO_2_ concentration with time between treatments in hypothetical regression lines although data were not analysed with time as a continuous variable; Fig 1A). The addition of lignocellulose alone (^13^C-CTRL treatment) did not have an effect on total respiration. The occurrence of PE was assessed by the difference in lignocellulose mineralisation between ^13^C-CTRL and diatom-amended treatments 1D and 2D. The effect of diatom addition on lignocellulose mineralisation depended on time (interaction between time and treatment; *F* = 3.58, d.f._6,20_, *p* = 0.014) (Fig 1B). Lignocellulose mineralisation was significantly higher in both 1D and 2D compared to ^13^C-CTRL on day 7 (*p*<0.001 in both cases). No difference was observed between ^13^C-CTRL and 1D between 14 and 28 days. During the same period, lignocellulose mineralisation in 2D was significantly higher compared to ^13^C-CTRL and 1D on days 14 and 28 (*p*<0.01 in both cases) but not on day 21. Within each treatment, lignocellulose mineralisation did not differ with time between 14 and 28 days. The amount of mineralised lignocellulose as a proportion of the total added material ranged between 7.0% in ^13^C-CTRL and 1D and 8.6% in 2D. For comparison, lignocellulose mineralisation rates were highest during the initial 7 days in the diatom treatments (2D>1D>^13^C-CTRL; 1.28, 1.05, 0.62 μg C-CO_2_ m^-1^_ws_ d^-1^ respectively) while the opposite was true for the period between 7 and 14 days (^13^C-CTRL>2D>1D; 0.83, 0.63, 0.45 μg C-CO_2_ m^-1^_ws_ d^-1^ respectively). Mineralisation rates of lignocellulose dropped to nearly zero in all treatments between 14 and 28 days.

### Incorporation of lignocellulosic C into microbial biomass

**Fig 2:**
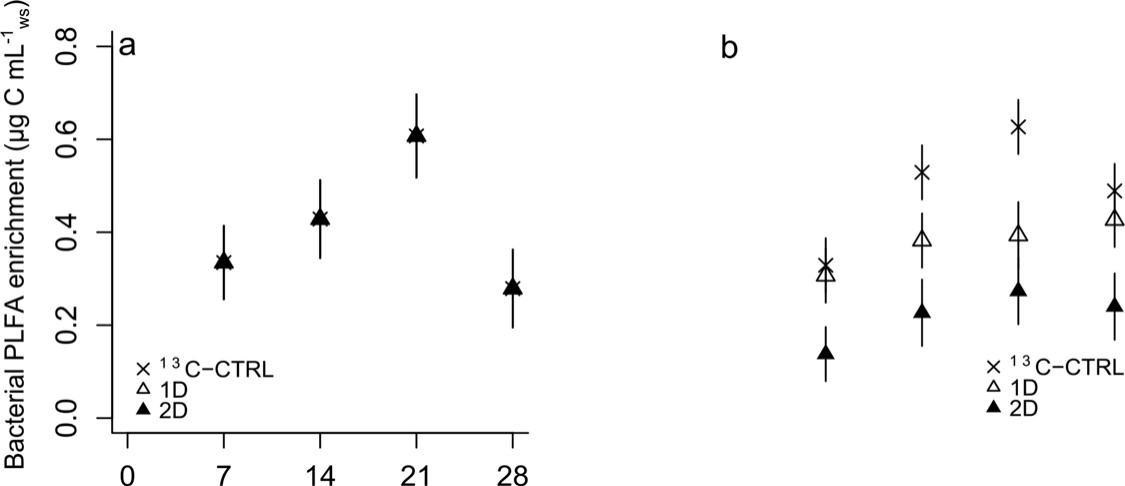
Incorporation of lignocellulosic carbon into bacterial (a) and eukaryotic (b) biomass with time and treatment based on the ^13^C enrichment of PLFA biomarkers. Data in (a) exactly overlap so that symbols for ^13^C-CTRL and 1D are hidden under the 2D filled triangles. Data represent the mean ± s.e.m. (*n* = 3).

The incorporation of lignocellulosic C into bacterial biomass did not differ between treatments but increased significantly with time (*F* = 10.60, d.f._3,29_, *p*<0.001) peaking at 21 days (Fig 2A). The enrichment pattern of the eukaryotic lignocellulose-responsive population was weakly dependent on time and treatment (*F* = 2.59, d.f._6,21_, *p* = 0.048), and enrichment levels were generally inversely related to diatom quantity (Fig 2B). Eukaryotic enrichment increased significantly in ^13^C-CTRL between 7 and 21 days but did not change with time in 1D and 2D.

### Microbial community structure

**Fig 3:**
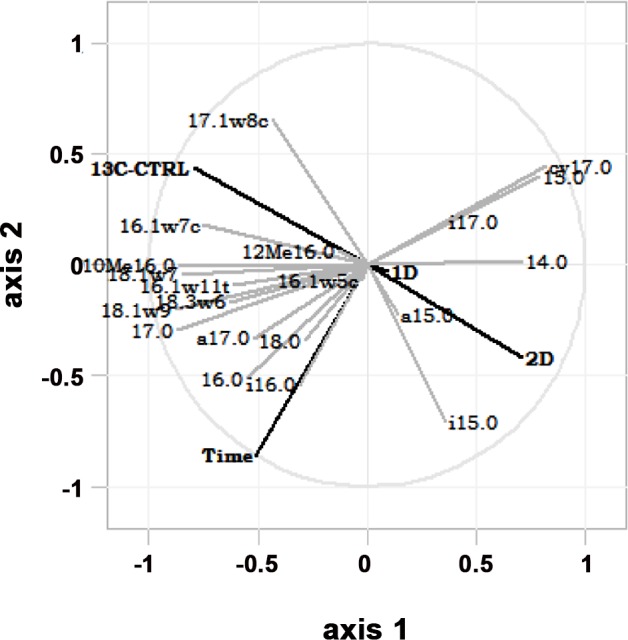
Partial least squares regression biplot (axes 1 and 2) showing differences in the proportional uptake of lignocellulosic carbon into PLFAs with time and treatment.

Axes 1 and 2 of the PLS model explained 49% of the variation in PLFA enrichment. The enrichment pattern of the lignocellulose-responsive community differed with diatom carbon availability; treatments ^13^C-CTRL and 2D were placed on opposite directions on the PLS biplot (Fig 3). 1D was aligned around the mid-point between ^13^C-CTRL and 2D, suggesting that 1D PLFA composition is intermediate on a continuum between the two more extreme treatments. The microbial community in ^13^C-CTRL was characterized by increased uptake of lignocellulosic carbon into monounsaturated PLFAs (16:1ω7, 17:1ω8, 18:1ω7, 18:1ω9) and 10Me16:0 (Fig 3). The enrichment of these fatty acids increased with time except for 17:1ω8. There was a strong positive association of 14:0 and i15:0 and the 2D treatment. Saturated PLFAs (16:0, 18:0), i16:0 and a17:0 increased while 15:0 and cy17:0 decreased with time regardless of treatment.

The PLS analysis compared the enriched PLFA profiles from this study with those of isolates known or suspected to degrade lignocellulose based on gene annotation. The analysis showed that the PLFA enrichment profiles from all treatments were highly correlated to members of the phylum Bacteroidetes (*Gramella*, *Flavobacterium*) and Gammaproteobacteria (*Teredinibacter turnerae*, *Simiduia* sp., *Marinomonas mediterranea*, *Cellvibrio japonicus*) on PLS axes 1 and 2 (S1 Fig). The analysis did not reveal dominance of a single microorganism since none of the isolates’ PLFA signature was concordant on all of the first 3 PLS axes. The PLFA signature of our microcosms is more likely to represent the average PLFA profile of a community of microbes involved in lignocellulose degradation, although a similar pattern could arise from the dominance of a microorganism not present in our dataset of isolates.

## Discussion

The addition of diatomaceous material produced a significant stepwise effect on total CO_2_ production reflecting the activation of an opportunistic saprophytic population with r-strategist characteristics that was able to rapidly metabolise the given labile substrate [44,45]. CO_2_ evolution rates in 1D and 2D declined after the surge of CO_2_ in the first week to background levels reflecting a drop in microbial activity as the most energy-rich compounds in the diatom substrate became depleted or transformed into more refractory forms. The short-term effect of diatom supply regardless of initial quantity is consistent with previous observations that marine bacteria are able to utilise labile compounds in less than 48 hours and produce refractory dissolved organic matter that may persist for more than a year [46,47].

Lignocellulose mineralisation was higher in diatom-amended treatments on day 7 coinciding with the phase of increased overall microbial activity and CO_2_ production due to diatom addition. Following the first sampling, the amount of mineralised lignocellulose in controls reached the same levels as in the low diatom treatment (1D), whereas it was not consistently higher in the high diatom treatment (2D). This inconsistency suggests that priming intensity was not high enough to clearly differentiate from variability due to natural heterogeneity between replicate slurries. The faster initiation of lignocellulose mineralisation in diatom-amended treatments could be attributed to the decomposition of accessible polysaccharide components within the lignocellulose complex by activated diatom degraders. The fact that we observed only differences in time and not overall quantity suggests a marginal role of PEs in lignocellulose degradation under the experimental conditions. This is further supported by the microbial utilisation of lignocellulosic carbon as demonstrated by PLFA biomarker enrichment; there was either no effect of treatment in the case of bacteria or a negative effect of the diatom substrate in the case of eukaryotic lignocellulose degraders. For comparison, van Nugteren et al. (2009) measured significant priming of bulk organic matter degradation 21 days after diatom addition in intertidal sediments under similar experimental conditions as here. The above suggest that lignocellulose degradation may be restricted even at conditions that favour priming of bulk sedimentary organic matter. A plausible explanation would be the inability of slow-growing lignocellulose degraders to compete with other members of the microbial community for access to labile substrates [48]. Alternatively the high N levels in the bulk sediment (C/N: 7.3) may not have promoted priming through the mechanism of “microbial mining” for N [49]. Recent studies have also failed to detect priming of pre-aged terrestrially-derived organic matter (C/N: >60) under non-limiting nutrient conditions in freshwater ecosystems using a, similar to ours, dual substrate approach [50,51] and of refractory marine dissolved organic matter by glucose addition [52]. On the other hand, the degradation of whole leaf litter (C/N: ~20) was primed at the presence of algae but only under low nutrient conditions [53]. All the above suggest that both the value of a substrate as a nutrient source for microbes and the level of external nutrients could regulate the occurrence of priming.

The degradation of lignocellulose is a complex process requiring the concerted action of several extracellular enzymes that only a limited number of microbes have evolved to produce [12,54]. A recent bioinformatic analysis of the phylogenetic distribution of cellulases in bacteria revealed that only 24% of bacterial sequenced genomes could be described as potential cellulose degraders encoding endo- and exo-cellulases [55]. We used the enrichment of PLFA biomarkers to evaluate the relative importance of bacteria and eukaryotes in lignocellulose degradation. The comparable temporal trends between bacterial and eukaryotic PLFA enrichment suggest that the latter is largely the result of direct utilisation of lignocellulosic carbon rather than predation on bacteria by microeukaryotes. Eukaryotic enrichment was negatively correlated to diatom quantity and maximised at the absence of diatoms. Although we did not observe a difference in the total bacterial biomass between treatments, it is safe to assume that more activated bacteria exist in diatom treatments based on mineralisation data. The above suggest that diatom-degrading bacteria could have suppressed eukaryotic growth. Negative effects of bacteria on fungal growth and biomass accrual have been previously observed in freshwater systems during plant litter decomposition [56]. The contribution of eukaryotes to lignocellulose degradation seemed to be considerably lower than that of bacteria. Earlier studies have also found higher efficiency of marine bacterial assemblages in lignocellulose degradation compared to fungi isolated from the same location [57,58]. The existing data thus support the hypothesis that environmental conditions may have allowed bacteria to dominate this ecosystem function in the marine environment suggesting fundamental differences in the function of terrestrial and marine ecosystems regarding lignocellulose degradation. It is noted however, that more sophisticated methods than PLFA-SIP or culture-based experiments may be necessary to unravel the full role of eukaryotes (e.g., fungi), as saprophytes in seawater and sediments.

Changes in the structure of the active lignocellulose-degrading community in different treatments were broadly assessed through multivariate analysis of relative PLFA enrichment. Although no difference was observed in the total bacterial enrichment between treatments, the multivariate analysis suggested the development of distinct lignocellulose-responsive populations at the presence of diatoms. The lignocellulose-responsive community in 2D was characterised by higher abundance of Gram-positive bacteria based on the higher enrichment of branched-chain PLFAs. In contrast, higher enrichment of monounsaturated fatty acids in ^13^C-CTRL suggested greater development of Gram-negative bacteria. Differences between treatments may reflect the contribution of certain diatom-degrading microbes in lignocellulose degradation in 2D either directly or indirectly by consumption of lignocellulose degradation by-products. Comparison of the PLFA enrichment profiles in this study with published PLFA profiles of known or suspected lignocellulose-degrading bacteria showed strong similarities with members of Bacteroidetes and Gammaproteobacteria (S1 Fig). Although PLFA analysis does not offer great power for phylogenetic identification, the same two phyla were identified as major bacteria of microbial biofilms on cellulose baits suspended in seawater using next generation sequencing [59]. Members of Gammaproteobacteria (Kangiella) and Bacteroidetes (Flavobacteria), as well as Spirochaetes and Deltaproteobacteria (Desulfosarcina) have also been identified as responsive to lignocellulose in salt-marsh sediments by DNA-SIP [60].

This study employed stable isotope labelling techniques to quantify the effect of priming on the mineralisation and utilisation of terrOC by sediment microbes. We used lignocellulose as a proxy for terrOC; it is acknowledged however that terrOC entering freshwater and marine environments is a heterogeneous mix of compounds consisting, apart from lignocellulose, of soil, petrogenic and black organic carbon [6]. Our results showed significant priming on lignocellulose mineralisation by diatom addition within the first week of the experiment however this effect was short-term and no difference in the total amount of respired lignocellulose could be detected between treatments by the end of the experiment. The faster initiation of lignocellulose mineralisation in diatom-amended treatments could be attributed to the decomposition of accessible polysaccharide components within the lignocellulose complex by activated diatom degraders. Differences in the timing of priming could be important under natural conditions where decomposition under aerobic conditions before burial in deeper anoxic sediments may be limited. Follow-on priming studies that allow microbial interactions within intact sediment matrices at a range of nutrient regimes and pulsed substrate additions will be necessary to fully appreciate the ecological significance of priming in marine benthic environments.

## Supporting Information

S1 Fig Comparison of the enriched PLFA dataset from this study with published PLFA profiles of known or suspected lignocellulose-degrading bacteria. (DOC)
