# Correction: Occurrence of Priming in the Degradation of Lignocellulose in Marine Sediments

**DOI:** 10.1371/journal.pone.0154365

**Published:** 2016-04-21

**Authors:** The PLOS ONE Staff

Fig 2 is incorrect. The axes are missing in panel b. The authors have provided a corrected version here. The publisher apologizes for the error.

**Fig 2:**
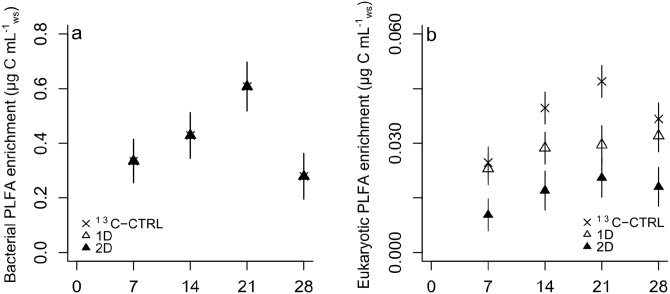
Incorporation of lignocellulosic carbon into bacterial (a) and eukaryotic (b) biomass with time and treatment based on the 13C enrichment of PLFA biomarkers. Data in (a) exactly overlap so that symbols for 13C-CTRL and 1D are hidden under the 2D filled triangles. Data represent the mean ± s.e.m. (n = 3).
