# Supplementary material for: Occurrence of Priming in the Degradation of Lignocellulose in Marine Sediments
Source: PLoS One. 2015 Dec 3;10(12):e0143917. doi: 10.1371/journal.pone.0143917 (PMC4669084; doi:10.1371/journal.pone.0143917)
Supplement: S1 Fig — (DOC) [file pone.0143917.s001.doc]

**Supporting Information**

**S1 Fig.** **Comparison of the enriched PLFA dataset from this study with published PLFA profiles of known or suspected lignocellulose-degrading bacteria or cultured representatives of bacterial genera that include strains encoding plant cell wall-degrading enzymes in their genome.** Bacterial genera in the database include members of Gammaproteobacteria [*Simiduia*^1^, *Saccharophagus degradans*^2^, *Teredinibacter turnerae*^2^, *Microbulbifer*^3^, *Shewanella* ^4^, *Colwellia* ^5^, *Pseudoalteromonas* ^6^, *Cellvibrio japonicus* ^7^, *Marinomonas mediterranea*^8^], Bacteroidetes [*Cytophaga hutshinsonii*^9^, *Cellulophaga*^10^, *Maribacter*^11,12,13^, *Gramella*^14,15,16^, *Flavobacterium*^17,18^, Firmicutes [*Bacillus aquimaris* (Yoon et al. 2003b)^19^, *Paenibacillus*^20^] and Actinobacteria isolated from the marine environment [*Isoptericola*^21,22^, *Streptomyces* ^23,24,25^, *Sciscionella marina*^26^, *Verrucosispora*^27^, *Salinispora*^28^]. Each biplot depicts the association between samples and explanatory variables on axes 1-2 (a) and 1-3 (b) respectively. PLS axes 1, 2 and 3 explained 90% of the variation in PLFA enrichment.

b


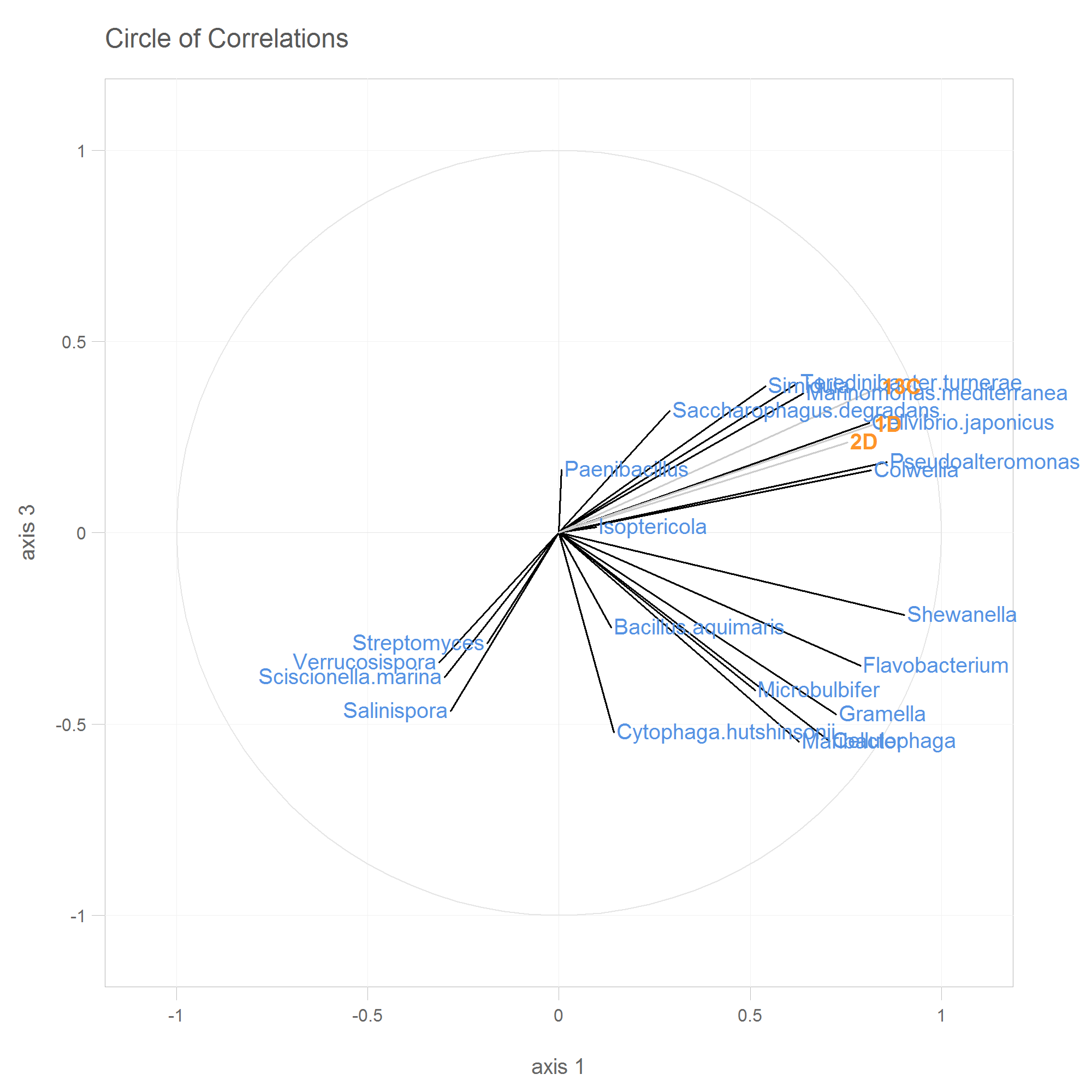


a

b

**S1 Fig references**

1. Shieh WY, Liu TY, Lin SY, Jean, WD & Chen J-S (2008) *Simiduia agarivorans* gen. nov., sp. nov., a marine, agarolytic bacterium isolated from shallow coastal water from Keelung, Taiwan. Int J Syst Evol Microbiol 58: 895-900.

2. Chen M-H, Sheu S-Y, Arun AB, Young C-C, Chen CA, Wang J-T,Chen W-M (2011) *Pseudoteredinibacter isoporae* gen. nov., sp. nov., a marine bacterium isolated from the reef building coral *Isopora palifera*. Int J Syst Evol Microbiol 61: 1887-1893.

3. Yoon J-H, Kim I-G, Shin D-Y, Kang KH,Park Y-H (2003a) *Microbulbifer salipaludis* sp. nov., a marine moderate halophile isolated from a Korean salt marsh. Int J Syst Evol Microbiol 53: 53-57.

4. Venkateswaran, K, Moser DP, Dollhopf ME, Lies DP, Saffarini DA, MacGregor BJ (1999) Polyphasic taxonomy of the genus *Shewanella* and description of *Shewanella* *oneidensis* sp. nov. Int J Syst Bacteriol 49: 705-724.

5. Bowman JP, Gosink JJ, McCammon SA, Lewis TE, Nichols DS, Nichols PD, Skerratt JH, Staley JT, McMeekin TA (1998)*Colwellia demingiae* sp. nov., *Colwellia hornerae* sp. nov., *Colwellia rossensis* sp. nov. and *Colwellia psychrotropica* sp. nov.: psychrophilic Antarctic species with the ability to synthesize docosahexaenoic acid (22:6ω3). Int J Syst Evol Microbiol 48: 1171-1180.

6. Ivanova EP, Zhukova NV, Svetashev VI, Gorshkova NM, Kurilenko VV, Frolova GM, Mikhailov VV (2000) Evaluation of phospholipid and fatty acid compositions as chemotaxonomic markers of *Alteromonas*-like proteobacteria. Cur Microbiol 41: 341-345.

7. Mergaert J, Lednicka D, Goris J, Cnockaert MC, De Vos P, Swings J (2003) Taxonomic study of *Cellvibrio* strains and description of *Cellvibrio ostraviensis* sp. nov., *Cellvibrio fibrivorans* sp. nov. and *Cellvibrio gandavensis* sp. nov. Int J Syst Evol Microbiol 53: 465-471.

8. Espinosa E, Marco-Noales E, Gómez D, Lucas-Elío P, Ordax M, Garcías-Bonet N, Duarte CM, Sanchez-Amat A (2010) Taxonomic study of *Marinomonas* strains from the seagrass *Posidonia oceanica*, with descriptions of *Marinomonas balearica* sp. nov. and *Marinomonas pollencensis* sp. nov. Int J Syst Evol Microbiol 60: 93-98.

9. Walker RW (1969) *Cis*-11-hexadecenoic acid from *Cytophaga hutchinsonii* lipids. Lipids 4(1): 15-18.

10. Bowman JP (2000) Description of *Cellulophaga algicola* sp. nov., isolated from the surfaces of Antarctic algae, and reclassification of *Cytophaga uliginosa* (ZoBell and Upham 1944) Reichenbach 1989 as *Cellulophaga uliginosa* comb. nov. Int J Syst Evol Microbiol 50: 1861-1868.

11. Nedashkovskaya OI, Kim SB, Han SK, Lysenko AM, Rohde M, Rhee M-S et al. (2004) *Maribacter* gen. nov., a new member of the family *Flavobacteriaceae*, isolated from marine habitats, containing the species *Maribacter aquivivus* sp. nov., *Maribacter orientalis* sp. nov. and *Maribacter ulvicola* sp. nov. Int J Syst Evol Microbiol 54: 1017-1023.

12. Yoon J-H, Kang S-J, Lee S-Y, Lee C-H, Oh T-K (2005) Maribacter dokdonensis sp. nov., isolated from sea water off a Korean island, Dokdo. Int J Syst Evol Microbiol 55: 2051-2055.

13. Barbeyron T, Carpentier F, L’Haridon S, Schüler M, Michel G, Amann R (2008) Description of *Maribacter forsetii* sp. nov., a marine *Flavobacteriaceae* isolated from North Sea water, and emended description of the genus *Maribacter*. Int J Syst Evol Microbiol 58: 790-797.

14. Nedashkovskaya OI, Kim SB, Lysenko AM, Frolova GM, Mikhailov VV, Bae KS et al. (2005) *Gramella echinicola* gen. nov., sp. nov., a novel halophilic bacterium of the family *Flavobacteriaceae* isolated from the sea urchin *Strongylocentrotus intermedius*. Int J Syst Evol Microbiol 55: 391-394.

15. Nedashkovskaya OI, Kim SB, Bae KS (2010) *Gramella marina* sp. nov., isolated from the sea urchin *Strongylocentrotus intermedius*. Int J Syst Evol Microbiol 60: 2799-2802.

16. Lau SCK, Tsoi MMY, Li X, Plakhotnikova I, Dobretsov S, Wong P-K, Qian P-Y (2005) *Gramella* *portivictoriae* sp. nov., a novel member of the family *Flavobacteriaeae* isolated from marine sediment. Int J Syst Evol Microbiol 55: 2497-2500.

17. Humphry DR, George A, Black GW, Cummings SP (2001) *Flavobacterium frigidarium* sp. nov., an aerobic psychrophilic, xylanolytic and laminarinolytic bacterium from Antarctica. Int J Syst Evol Microbiol 51: 1235-1243.

18. Nogi Y, Soda K, Oikawa T (2005) *Flavobacterium frigidimaris* sp. nov., isolated from Antarctic seawater. Syst Appl Microbiol 28: 310-315.

19. Yoon J-H, Kim I-G, Kang KH, Oh T-K, Park Y-H (2003b) *Bacillus marisflavi* sp. nov. and *Bacillus aquimaris* sp. nov., isolated from sea water of a tidal flat of the Yellow Sea in Korea. Int J Syst Evol Microbiol 53: 1297-1303.

20. Lee H-W, Roh SW, Yim KJ, Shin N-R, Lee J, Whon TW et al. (2013) *Paenibacillus marinisediminis* sp. nov., a bacterium isolated from marine sediment. J Microbiol 51(3): 312-317.

21. Zhang Y-Q, Schumann P, Li W-J, Chen G-Z, Tian X-P, Stackebrandt E et al. (2005) *Isoptericola halotolerans* sp. nov., a novel actinobacterium isolated from saline soil from Qinghai Province, north-west China. Int J Syst Evol Microbiol 55: 1867-1870.

22. Wu Y, Li W-J, Tian W, Zhang L-P, Xu L, Shen Q-R, Shen B (2010) *Isoptericola jiangsuensis* sp. nov., a chitin-degrading bacterium. Int J Syst Evol Microbiol 60: 904-908.

23. Zhao XQ, Li W-J, Jiao W-C, Li Y, Yuan W-J, Zhang Y-Q et al. (2009) *Streptomyces xinghaiensis* sp. nov., isolated from marine sediment. Int J Syst Evol Microbiol 59: 2870–2874.

24. Tian X-P, Zhang Y-Q, Li Q-X, Zhi X-Y, Tang S-K, Zhang S, Li W-J (2009a) *Streptomyces nanshensis* sp. nov., isolated from the Nansha Islands in the South China Sea. Int J Syst Evol Microbiol 59: 745–749.

25. Tian X-P, Xu Y, Zhang J, Li J, Chen Z, Kim C-J et al. (2012) *Streptomyces oceani* sp. nov., a new obligate marine actinomycete isolated from a deep-sea sample of seep authigenic carbonate nodule in South China Sea. Antonie van Leeuwenhoek 102: 335–343.

26. Tian X-P, Zhi X-Y, Qiu Y-Q, Zhang Y-Q, Tang S-K, Xu L-H et al. (2009b) *Sciscionella marina* gen. nov., sp. nov., a marine actinomycete isolated from a sediment in the northern South China Sea. Int J Syst Evol Microbiol 59: 222–228.

27. Dai H-Q, Wang J, Xin Y-H, Pei G, Tang S-K, Ren B. et al. (2010) *Verrucosispora sediminis* sp. nov., acyclodipeptide-producing actinomycete from deep-sea sediment. Int J Syst Evol Microbiol 60: 1807–1812.

28. Ahmed L, Jensen PR, Freel KC, Brown R, Jones AL, Kim BY,Goodfellow M (2013) *Salinispora pacifica* sp. nov., an actinomycete from marine sediments. Antonie van Leeuwenhoek 103: 1069–1078.
